# Subclinical Vitamin C Plasma Levels Associated with Increased Risk of CAD Diagnosis via Inflammation: Results from the NHANES 2003–2006 Surveys

**DOI:** 10.3390/nu15030584

**Published:** 2023-01-22

**Authors:** Jennifer M. Crook, Saun-Joo L. Yoon, Oliver Grundmann, Ann Horgas, Versie Johnson-Mallard

**Affiliations:** 1 Center for Health Equity and Community Engagement Research, Mayo Clinic, Jacksonville, FL 32224, USA; 2 College of Nursing, University of Florida, Gainesville, FL 32611, USA; 3 College of Pharmacy, University of Florida, Gainesville, FL 32611, USA; 4 College of Nursing, Kent State University, Kent OH 44242, USA

**Keywords:** CAD, heart disease, inflammation, nutritional surveillance, vitamin C

## Abstract

Vitamin C remains an important, yet frequently unassessed, component of a healthy immune system though it may prove useful in alleviating the chronic inflammatory processes underlying chronic diseases such as coronary artery disease (CAD). Recent research identified a sizeable proportion of the United States population with insufficient vitamin C plasma levels and significant associations to both acute and chronic inflammation. This cross-sectional study used the 2003–2006 NHANES surveys data to extrapolate associations between plasma vitamin C levels (deficiency, hypovitaminosis, inadequate, adequate, and saturating) and CAD through inflammation (C-reactive protein and red cell distribution width). Increased reports of CAD diagnosis were identified in participants with vitamin C deficiency (OR: 2.31, CI: 1.49–3.58) and inadequate plasma levels (OR: 1.39, CI: 1.03–1.87). No significant correlation was identified between any other plasma vitamin C quintiles and CAD. When inflammation was controlled, previous associations in the deficient level of plasma vitamin C were no longer significant in association with CAD and participants with inadequate plasma vitamin C showed a reduced association to CAD diagnoses (OR: 0.33, CI: 0.13-0.86). Most chronic inflammation and vitamin C plasma statuses do not demonstrate specific signs or symptoms until the deficient level of vitamin C and/or disease. Thus, increased surveillance of both, and healthy nutritional habits remain crucial modifiable risk factors for disease prevention.

## 1. Introduction

The anti-inflammatory and immune-boosting properties of vitamin C are historically well established [1,2]. It is known to enhance immune cell transport and functions while simultaneously lessening overactive immune responses [3]. Multiple benefits of vitamin C include collagen synthesis [4], reusing and enhancing other vitamins and minerals [5], and influencing epigenetic processes [6]. The ability of vitamin C to donate electrons explains many of its important functions [7]. More recently, the use of intravenous vitamin C has gained visibility for its effectiveness in coronavirus-19 (COVID-19) treatments [3,8,9]. Severe, including fatal, COVID-19 infections are associated with vitamin C deficiencies [10]. It is a water-soluble vitamin with limited storage capability in the body and is usually tolerated well in both oral and intravenous intakes. However, because humans are unable to synthesize vitamin C, they must rely on a consistent dietary intake. 

But what is a consistent dietary intake? Assessments of vitamin C dietary consumption and/or plasma status in annual examinations or on admission to inpatient settings are scarce. Patient dietary reports may prove unreliable and are usually uncorroborated with plasma levels [11]. The specific consequences of insufficient short-term and long-term dietary intake of vitamin C prior to a deficiency (scurvy) diagnosis remains unclear. Many clinical trials examining vitamin C supplementation benefits lack a baseline plasma status of the individual, which leads to inconclusive results, as research has identified a distinct cutoff where extra supplementation is needed to reverse a hypovitaminosis C status (11–23.99 μmol/L) [12]. 

Coronary artery disease (CAD) is known to possess both chronic inflammatory and autoimmune components, mainly due to atherosclerosis [13]. Like visceral adipocytes [14], accumulation of fatty acids in the vascular spaces stimulates inflammatory cascades and results in endothelial changes and chronic inflammation. Low-density lipoproteins (LDLs) that transport cholesterol through oxidative modifications were specifically identified to stimulate inflammatory responses, causing endothelial injury from the recruitment of T cells and macrophages and resulting in macrophage-derived foam cell generation [15]. These foam cells are responsible for the secretion of pro-inflammatory interleukin-6 (IL-6) and tumor necrosis factor-alpha (TNF-α) [15,16,17,18]. Also associated with CAD are hypertension, obesity, smoking, and decreased plasma vitamin C levels [19,20,21,22].

The epigenetic properties of vitamin C may explain its importance in prevention and treatment of CAD. Vitamin C and its 2-electron oxidation product, dehydro-L-ascorbic acid, have been identified to provide considerable benefit in CAD through a variety of mechanisms of action, including inhibiting LDL oxidation through binding to copper [23], assuaging baroreflex dysfunction to alleviate hypertension [24], restoring endothelial dysfunction through increased synthesis and deposition of collagen, promoting endothelial proliferation, inhibiting apoptosis, and free radical scavenging [25]. Epigenetic influencers in atherosclerosis include a TET2 enzyme histone modification that is epigenetic mark–specific, and microRNA and long noncoding RNA regulations [26,27,28], of which vitamin C has exhibited influence [29,30,31]. The pro-inflammatory cytokines IL-6, TNF-α, and high-sensitivity C-reactive protein (CRP) have been identified as being under epigenetic modification control [32,33], where vitamin C has also been proven beneficial [34,35,36].

Our previous work identified a large percentage of the United States (U.S.) population with insufficient levels of plasma vitamin C [11] prior to the COVID-19 pandemic. Additionally, those insufficient levels were associated with acute and chronic inflammation through CRP and red cell distribution width (RDW) levels [37]. Nationwide assessments of vitamin C status are inconsistent, and the current vitamin C status of U.S. residents remains unknown. With COVID-19 infections and pandemic stressors, such as quarantines, food insecurity, school closures, and supply chain issues, it is unclear how the nutritional status, including plasma vitamin C, of the population has been affected. Because of its inflammatory advantages in both acute and chronic inflammation [37,38,39], the benefits of a consistent sufficient plasma status may be realized in many disease processes, and an insufficiency of vitamin C explored.

The purpose of this study was to determine the association of five defined quintiles of vitamin C (deficiency, hypovitaminosis, inadequate, adequate, and saturating) to CAD and identify if those associations existed when types of inflammation (CRP and RDW) were controlled.

## 2. Materials and Methods

### 2.1. Data Source

This study used data from the 2003–2006 National Health and Nutrition Examination Surveys (NHANES). The complex, multistage sampling design of these nationally representative annual surveys and instructions for calculating sample weight construction can be found on the Centers for Disease Control and Prevention (CDC) website [40]. 

### 2.2. Participants

The sample selection pathway for this study, as well as the demographic variable descriptions, have been previously published [11]. Although the NHANES have been updated in recent years to be more inclusive of racial/ethnic differentiations, the variables of race/ethnicity in the surveys included in this study included Non-Hispanic White, Non-Hispanic Black, Mexican American, Other Hispanic and Other. From the NHANES surveys completed and published for the years 2003–2004 and 2005–2006, this study cohort included a final sample size of 7607 unique participants. Initially, all participants >18 years of age were to be included in the final dataset; however, inconsistencies with inclusion criteria in the Medical Conditions Questionnaire Data Set were identified. One survey excluded participants <20 years of age from participating in this portion of the survey, and one survey included adults ≥18 years of age, so initial plans to include all adults ≥18 years of age were modified to keep only participants who had complete data on all independent and dependent variables. Inclusion criteria for this study included all genders, ethnicities, and non-institutionalized adults ≥20 years of age willing and able to give informed consent who participated in both questionnaire and laboratory assessment measurements. Excluded were children, individuals in the military, and participants with incomplete data. 

### 2.3. Measurement of Plasma Vitamin C

Mobile examination clinics were used for specimen blood collection, where minimal processing was done prior to shipment to remote laboratories for assay processing. All variables analyzed in this study were taken from NHANES data collection that was made publicly available on their website in the 2003–2004 and 2005–2006 surveys. It should be noted that blood level values used in this study were not fasting laboratory values. Detailed information regarding the laboratory collection, processing, and reporting of the NHANES survey variables used in this study can be found on the CDC website [40]. 

Plasma ascorbic acid (vitamin C) was collected and measured by isocratic high-performance liquid chromatography with electrochemical detection at 650 mV. Peak area quantitation was then based on a standard curve generated from three different concentrations of an external standard (0.025, 0.150, and 0.500 mg/dL). The quality assurance and quality control protocols utilized by NHANES met the 1988 Clinical Laboratory Improvement Act mandates, and a full description of the specimen collection, laboratory processing method, and quality control procedures for vitamin C can also be found on the CDC website [40].

### 2.4. Measurement of Coronary Artery Disease (CAD)

Information on CAD was collected during home visits and included in the Medical Conditions section of the Questionnaire Data Set. Study participants could answer “yes” or “no” if they had ever been diagnosed with CAD to the following five questions: (1) Have you ever been told you had CHF (congestive heart failure)? (2) Have you ever been told you had CAD? (3) Have you ever been told you had angina? (4) Have you ever been told you had had a heart attack? (5) Have you ever been told you had a stroke? Interaction effects were seen between four of the five questions (CHF, CAD, heart attack, and stroke) and the independent variable of vitamin C recoded into quintiles. No interaction effects were seen between participant reports of angina diagnoses; thus, a diagnosis of angina was excluded from recoding of the CAD variable. For this study, the NHANES variables of CHF, CAD, heart attack, and stroke were combined to form a new variable that was retained as dichotomous (yes/no).

### 2.5. Covariates

To analyze inflammation, the biomarkers CRP and RDW were examined. CRP levels were analyzed with latex-enhanced nephelometry, and particle-enhanced assays were used for quantitation. A Behring nephelometer was used to perform assays and determine quantitative CRP levels with the primary standard organized by Behring Diagnostics and standardized against WHO reference material [41]. More detailed laboratory processing information can be found on the CDC website [41]. RDW was captured in the complete blood cell count (CBC) and processed via a Beckman Coulter MAXM Instrument deriving CBC parameters based on the Beckman Coulter method of counting, sizing, automatic diluting and mixing for sample processing, and using a single beam photometer for hemoglobinometry [41]. Additional information regarding the processing of CBC with differential specimens can be found on the CDC website [41]. 

Confounding variables were selected based on both relevance in previous studies in the literature and having been captured in the NHANES 2003/2004 and 2005/2006 surveys. Food security [42,43] was assessed in the Food Security Questionnaire. Respondents answered “yes” or “no” to the question, “Are you worried you will run out of food?” Smoking status [44,45] information was included in the Smoking Recent Tobacco Use Questionnaire where respondents answered “yes” or “no” to the question, “Used tobacco/nicotine in the last 5 days?” The variable of Poverty to Income Ratio was collected in the Demographic Questionnaire and calculated using Department of Health and Human Services’ poverty guidelines for determining qualification for financial assistance for federal aid programs such as Food Stamps, Women, Infants, and Children (WIC), and the National School Lunch Program [23]. This variable provides much more context than poverty alone, which has been associated with both increased cardiac mortality and decreased vitamin C status [46,47]. The variable was recoded into categorical values of: High PIR (participants below 30% of the poverty line eligible for government assistance, or ≤$25,000 per year), Medium PIR (considered middle class with a household income of $25-$75,000 per year), and Low PIR (considered high-income earners with household incomes of >$75,000 per year). Comorbidity diagnoses of hypertension [48,49], diabetes [50], hypercholesterolemia [51,52], rheumatoid arthritis [53,54,55], and asthma [56,57,58] were also included. 

No correlations were identified in the initial analysis between the continuous vitamin C variable and other tested variables. The vitamin C variable was recoded into the following five categories based on participant plasma levels: deficiency (0–10.99 μmol/L), hypovitaminosis (11–23.99 μmol/L), inadequate (24–49.99 μmol/L), adequate (50–69.99 μmol/L), and saturating (≥70 μmol/L). Although there are minimal variations in most definitions of hypovitaminosis, inadequate, adequate, and saturating levels, the parameters for the ranges used in this study were derived from studies examining hypovitaminosis and supplementation, as well as the saturating levels in which maximum immune support was achieved [12,59].

### 2.6. Statistical Analysis

Data from the 2003–2006 NHANES data sets were downloaded in a Statistical Analysis System (SAS) transport file format, version 9.4 (SAS Institute Inc., Cary, NC, USA). SAS files were converted to Statistical Package for the Social Sciences files (SPSS) version 26.0 (IBM SPSS Statistics for Windows, Armonk, NY, USA) for analysis. Four-year sample weights were calculated per the National Center for Health Statistics guidelines. Descriptive statistics were used to examine plasma vitamin C quintiles with Pearson’s chi-square tests applied for categorical variables. Analysis of variance (ANOVA) tests were utilized for continuous variables. In all statistical tests, a *p* value less than 0.05 was considered statistically significant. Proportion percentages with standard deviations (SD) were reported for categorical variables, while means, percentages, and frequencies with SD were used to present continuous variables.

To determine the relationship between plasma vitamin C quintiles and CAD, while controlling for inflammation (CRP and RDW), unadjusted and fully adjusted logistic regression models were created. Assumptions of the logistic regression, including the independence of errors, absences of multicollinearity, and no strong influential outliers were assessed and found to be met. The sample was weighted according to NHANES guidelines on the CDC website as described previously, and interaction effects between the dependent variable, CAD, were independently compared to all covariates. Significance was set at *p* ≤ 0.05, and any interactions assessed. The adjusted model included *a priori* confounders of hypertension, hypercholesterolemia, diabetes, asthma, and rheumatoid arthritis, as well as the inflammatory biomarkers CRP and RDW. Collinearity diagnostics were performed for continuous variables, and the assumption of no collinearity was found to be met. Hosmer–Lemeshowe tests determined the regression models to be good fits. Nagelkerke R-square tests calculated the amount of CAD variation that could be explained by vitamin C plasma levels. Finally, the extent to which each category of the plasma vitamin C level was able to predict the outcome of a positive diagnosis of CAD was interpreted as an adjusted odds ratios (aOR) with 95% confidence intervals (CI).

## 3. Results

Most study participants (40.1%) were of middle-adulthood age (40–59 years of age), female (51.3%), nonsmokers (70.6%), and food secure (85.9%). Many were non-Hispanic White participants (73.6%) with “high” poverty-to-income ratio (PIR) levels or in high-income households (63.8%). Many had reported no diagnoses of hypertension (69.9%), diabetes (91.2%), hypercholesterolemia (58.8%), or rheumatoid arthritis (83.1%), though a larger proportion of respondents with asthma (58.9%) was identified. Participants displayed a mean BMI of 28.68 ± 6.44 kg/m^2^, plasma vitamin C of 54.63 ± 28.62 μmol/L, plasma CRP of 0.48 ± 0.92 mg/dL, and RDW of 12.86 ± 1.2%. Most participants reported no diagnosis of CAD (93.1%). Table 1 includes demographic information of the study sample.

Table 2 examines more closely, the characteristics of the sample within the five defined quintiles of plasma vitamin C. Males possessed significantly less saturating (19.3%) and more inadequate (28.7%) vitamin C plasma levels when compared to females. Young adult participants displayed the least deficiency (3.4%), hypovitaminosis (8.1%), and inadequate (29.9%) levels of all age group categories. Race/ethnicity variables showed significantly higher proportions of Mexican American, Other Hispanic, and Non-Hispanic Black inadequate vitamin C levels (30.8%, 30.0%, and 32.4%, respectively). PIR showed wide disparities of high saturating vitamin C (33.9%) and low deficient levels (3.0%) in the highest income-earning households (low PIR). Participants who admitted to food insecurity showed significantly lower proportions of adequate (29.1%) and saturating (17.3%) plasma vitamin C compared to those with no food issues. Hypertension showed increased proportions in all insufficient vitamin C quintiles (deficient, hypovitaminosis, and inadequate) and decreased proportions in sufficient quintiles (adequate and saturating). Participants with diabetes displayed significantly higher inadequate vitamin C levels (31.1%) and lower saturating levels (20.0%) than participants without (25.5% and 26%, respectively). Asthma, hypercholesterolemia, and rheumatoid arthritis did not show significant differences across the vitamin C quintiles. Means of BMI, CRP, and RDW, however, were found to be statistically significant. Respondents who had been diagnosed with CAD showed lower proportions of sufficient (adequate and saturating) levels of vitamin C when compared to those without CAD diagnoses.

Two logistic regression models were generated to identify associations of plasma vitamin C quintiles to participant reports of CAD diagnosis (see Table 3). In the unadjusted model, plasma vitamin C (χ^2^ = 4.11, df = 4, *p* = 0.003) quintiles were significantly associated with CAD diagnosis. Wald χ^2^ goodness-of-fit test results identified the following variables as significant predictors of CAD: gender (χ^2^ = 5.91, df = 1, *p* = 0.02), age (χ^2^ = 157, df = 2, *p* < 0.001), race/ethnicity (χ^2^ = 2.63, df = 3.2, *p* = 0.04), food security (χ^2^ = 26.1, df = 1, *p* < 0.001), and BMI (χ^2^ = 9.30, df = 1, *p* = 0.02). Smoking status (χ^2^ = 0.61, df = 1, *p* = 0.43) and PIR (χ^2^ = 4.97, df = 2, *p* = 0.08) were not found to be significant predictors of CAD within this sample. At increased risk of CAD diagnosis were men, middle- and late-adulthood participants, food insecure, and respondents with vitamin C inadequate and deficient plasma levels. Presenting with decreased risk of CAD diagnosis as compared to White, Non-Hispanic participants were those of Mexican American race/ethnicity.

After adjusting for inflammation and other potential confounders, a significant association between only the inadequate plasma vitamin C quintile and CAD was identified (χ^2^ = 10.0 df = 4, *p* = 0.04) with a now reduction in the odds of reporting CAD (OR: 0.33, 95% CI 0.13–0.86). Significant predictor variables in the adjusted model included race/ethnicity (χ^2^ = 11.6, df = 4, *p* = 0.02), food insecurity (χ^2^ = 10.7, df = 1, *p* = 0.001), diabetes (χ^2^ = 8.56, df = 1, *p* = 0.003), hypertension (χ^2^ = 6.81, df = 1, *p* = 0.009), hypercholesterolemia (χ^2^ = 3.72, df = 1, *p* = 0.05), BMI (χ^2^ = 9.85, df = 1, *p* = 0.002), and RDW (χ^2^ = 6.60, df = 1, *p* = 0.01). Previously identified associations within the variables of gender, Mexican American race/ethnicity, and inadequate plasma vitamin C levels were no longer presented. With inflammation and confounders controlled, new associations to CAD diagnoses were identified between Other Hispanic, Non-Hispanic Black and Other race/ethnicity participants. Food insecurity retained a statistically significant risk of CAD diagnoses. Participants with diabetes presented with 3.14 times increased odds of CAD (95% CI: 1.40–7.07) after controlling for inflammation. Only the inflammatory biomarker RDW retained a statistically significant association to CAD diagnosis in the adjusted model. 

## 4. Discussion

Chronic inflammation remains a challenging issue to explore. There are currently no suggested parameters of clear laboratory markers to assess, surveil, or mark progress. Likewise, nutritional status, especially regarding vitamin C, is largely ignored until symptoms of deficiency arise. Because of the anti-inflammatory, antioxidant, and immune-boosting properties of vitamin C [60] it is plausible that chronic insufficient vitamin C due to both inadequate nutritional intake and increased bodily consumption contributes to the progression of chronic diseases with inflammatory components, such as CAD. Maintaining a sufficient level of vitamin C may be beneficial in the prevention of such diseases.

In this study, individuals with deficient and inadequate plasma vitamin C levels exhibited significantly increased odds of CAD diagnosis. Participants with vitamin C deficiency were 2.31 times more likely to report a CAD diagnosis than those with saturating plasma levels (95% CI: 1.49–3.58). Persons possessing inadequate plasma levels of vitamin C were 1.39 times more likely to report a CAD diagnosis (95% CI: 1.03–1.87). No significant correlation was identified for any other plasma vitamin C quintile and CAD. However, when inflammation (CRP and RDW) was controlled, a previously identified association between deficient vitamin C plasma levels and CAD disappeared, and the odds for a CAD diagnosis were significantly reduced in inadequate plasma vitamin C participants, supporting the hypothesis that one plausible explanation of inflammation in CAD is insufficient levels of plasma vitamin C [61]. These findings provide a glimpse into the importance of maintaining a consistent, adequate level of vitamin C to lessen the risk of developing CAD and reveal the need for increased surveillance and/or supplementation of both nutritional and chronic inflammation status. Though risk factors for the development of CAD encompass more than nutrition [62], the findings from this study warrant closer nutritional and inflammatory surveillance beyond patient report.

Food security was identified to be associated with greater odds of a CAD diagnosis. The recent COVID-19 pandemic has increased disparities in health access and food security by almost double the amount pre-pandemic (currently estimated to be as high as 921 million food-insecure people) [63,64,65]. Nutritional insufficiencies and the resulting sequalae of disease due to inadequate diets and increased inflammation is, and will remain, a worldwide crisis. Thus, it is suggested that both be explored further on other nutritional insufficiencies known to be associated with inflammatory triggers.

Insufficient levels of vitamin C (defined here as deficiency, hypovitaminosis, and inadequate), specifically inadequate plasma levels (24–49.99 μmol/L), were previously identified to be associated with both acute and chronic inflammatory biomarkers [37], though they may not present with specific signs or symptoms of a problem. Likewise, the levels of inflammation seen in this study within CRP and RDW are not clinically indicative of specific inflammation or above current normal standards, compounding the importance of the need for chronic inflammatory assessment guidelines for surveillance and treatment. Currently, vitamin C is not readily captured during routine assessments, though research is elucidating technological advances to increase accessibility and affordability for hospitals and clinician use [66]. Patient report of dietary intake has been shown to not corroborate with plasma vitamin C levels [11], so it is recommended that future research continue to identify laboratory processes that allow clinicians to assess nutritional status in both inpatient and outpatient settings.

Limitations of this study include the inability to define the original variables captured in a dataset and used for secondary analysis. Physical activity, though well established as associated with plasma vitamin C [67,68,69,70], was not included in this study due to the limited scope of the NHANES in capturing exercise. Questionnaires capturing participant reports of CAD differed between the 2003–2004 and 2005–2006 studies (participants >18 years of age were involved in one survey and subjects >20 years of age were included in the subsequent survey), which excluded the use of adult-aged participants 18 and 19 years of age. This age group would have benefited from inclusion, as they have reached the age of independence to establish their own dietary patterns. Other recognized potential variables related to inflammation, CAD, and/or vitamin C, such as allergies, the use of anti-inflammatory medications, and sickle cell anemia, among others, were not captured in these NHANES surveys. Future research is recommended to explore the significance of associations more fully with the provided vitamin C plasma categories defined here. The use of a nationally representative survey that examined the nutritional health of the U.S. population was not specifically sampling participants with CAD, so the sample numbers were greatly reduced when compared to the study population, possibly excluding a true association. Another significant limitation includes the lack of inclusion of inflammatory biomarkers IL-6 and TNF-α variables and other biomarkers, including hs-CRP which have been identified to be a more accurate diagnostic indicator of inflammation than CRP [71]. Limitations inherent in using cross-sectional studies include the inability to determine cause and effect, participant and recall bias, the inability to identify the amount of time since diagnosis, and the behavioral changes that may accompany such a diagnosis, including dietary and activity modifications that may hide true associations between insufficient plasma vitamin C and CAD. 

RDW was found to be significant in the adjusted logistic regression model for CAD, but not CRP. This finding directs attention more to chronic inflammation present within the population and supports the recommendation to identify use of the easier and more cost-effective CBC test for RDW results as a diagnostic indicator of inflammation. RDW, a count of the size variability in circulating erythrocytes, continues to be exposed as a reliable indicator of chronic inflammation, as it may highlight a disturbance in homeostasis during the 120-day life cycle of red blood cells. RDW has been associated with CRP and multiple chronic diseases with inflammatory processes [72,73,74,75]. RDW has also been associated with insufficient levels of vitamin C [37].

A substantial increase in the odds of CAD was identified in participants with inadequate and deficient plasma levels of vitamin C. When inflammation was controlled, the previously identified increased odds in both of those vitamin C quintile categories disappeared and individuals with inadequate vitamin C plasma levels were then discovered to possess a reduced likelihood of reporting a CAD diagnosis. Future efforts with more robustly designed randomized control trial studies would be beneficial to confirm the association between all insufficient levels (deficient, hypovitaminosis, and inadequate) of vitamin C and targeted CAD interventions. It is also recommended that research direct efforts to the effects of poor nutritional status and unchecked chronic inflammation with suggested prospective cohort and interventional studies. The sample in this study was not specific to cardiac diseases, and although the literature has revealed initial evidence of inflammatory processes common in CAD instigation and progression, more conclusive confirmation can only be obtained from stronger study designs. COVID-19 has exposed wide disparities in food access and increased the peripheries of food deserts. Results of this study suggest that the short- and long-term effects of nutritional insufficiencies of vitamin C and its mitigating effect on inflammation should be further examined. Because it is solely diet-dependent, population insufficiencies of vitamin C imply other nutritional deficits that may also contribute to unregulated inflammation.

## Figures and Tables

**Table 1:** Sample Description (*n* = 7607) in NHANES 2003–2006 Surveys.

Characteristics	Sample (*n* = 7607)	Weighted Sample by %	Mean (SD)
Gender Male Female	3699 3908	48.7 ± 0.5 51.3 ± 0.5	
Adulthood age, y Young, 20–39 Middle, 40–59 Late, ≥60	2751 2295 2561	36.5 ± 0.8 40.1 ± 0.8 22.3 ± 0.5	
Race/Ethnicity Mexican American Other Hispanic Non-Hispanic White Non-Hispanic Black Other	1516 230 4305 1536 290	7.6 ± 1.1 3.4 ± 0.5 73.6 ± 2.1 10.5 ± 1.2 4.9 ± 0.4	
Family PIR High (0–1.5) Medium (1.51–4.5) Low (>4.51)	5206 1614 787	63.8 ± 1.1 22.7 ± 0.5 13.5 ± 0.6	
Smoking status Yes No	3392 5610	29.4 ± 1.0 70.6 ± 1.0	
Food insecurity Yes No	1449 6158	14.1 ± 0.8 85.9 ± 0.8	
Hypertension Yes No	2528 5052	30.1 ± 0.6 69.9 ± 0.6	
Asthma Yes No	585 380	58.9 ± 1.9 41.1 ± 1.9	
Hypercholesterolemia Yes No	2270 3051	41.2 ± 0.8 58.8 ± 0.8	
Diabetes Yes No	892 6715	8.8 ± 0.4 91.2 ± 0.4	
Rheumatoid Arthritis Yes No	401 1617	16.9 ± 1.0 83.1 ± 1.0	
BMI, kg/m^2^			28.88 (6.6)
Vitamin C, μmol/L			42.44 (8.3)
CRP, mg/dL			0.53 (1.0)
RDW			12.91 (1.3)
CAD Yes	701	6.9 ± 0.5	
No	6906	93.1 ± 0.5	

Abbreviations: PIR, Poverty to Income Ratio; BMI, body mass index; CAD, coronary artery disease; CRP, C-reactive protein; PIR, poverty to income ratio; RDW, red cell distribution width.

**Table 2:** Baseline Characteristics of Participants by Plasma Vitamin C Status.

Characteristics	Plasma Vitamin C concentration (μmol/L)						
	Total	Deficiency (0–10.99 μmol/L)	HYPOVITAMINOSIS (11–23.99 μmol/L)	Inadequate (24–49.99 μmol/L)	Adequate (50–69.99 μmol/L	Saturating (≥ 70 μmol/L)	*p* Value
Vitamin C, μmol/L	7607	6.85 ± 2.78	17.5 ± 3.69	38.3 ± 7.37	38.3 ± 5.77	89.8 ± 21.9	0.00
Gender Male Female	3699 3908	307 (8.3%) 160 (4.1%)	425 (11.5%) 297 (7.6%)	1062 (28.7%) 929 (23.8%)	1191 (32.2%) 1276 (32.7%)	714 (19.3%) 1246 (31.9%)	<0.001
Adulthood Category Young, 20–39 Middle, 40–59 Late, ≥60	2751 2295 2561	50 (3.4%) 82 (6.4%) 101 (7.8%)	118 (8.1%) 131 (10.2%) 139 (10.7%)	425 (29.0%) 386 (30.0%) 393 (30.2%)	525 (35.9%) 421 (32.7%) 424 (32.6%)	345 (23.6%) 268 (20.8%) 245 (18.8%)	<0.001
Race/Ethnicity Mexican American Other Hispanic Non-Hispanic White Non-Hispanic Black Other	1516 230 4305 1536 290	71 (7.2%) 4 (1.7%) 290 (7.2%) 92 (6.0%) 10 (3.4%)	123 (8.1%) 23 (10.0%) 403 (10.0%) 139 (9.0%) 34 (11.7%)	467 (30.8%) 69 (30.0%) 880 (21.8%) 497 (32.4%) 78 (26.9%)	546 (36.0%) 86 (37.4%) 1226 (30.4%) 505 (32.9%) 104 (35.9%)	309 (20.4%) 48 (20.9%) 1236 (30.6%) 303 (19.7%) 64 (22.1%)	<0.001
Family PIR High (0–1.5) Medium (1.51–4.5) Low (>4.51)	5206 1614 787	323 (6.2%) 120 (7.4%) 24 (3.0%)	520 (10.0%) 146 (9.0%) 56 (7.1%)	1424 (27.4%) 395 (24.5%) 172 (21.9%)	1690 (32.5%) 509 (31.5%) 268 (34.1%)	1249 (24.0%) 444 (27.5%) 267 (33.9%)	<0.001
Smoking status Yes No	3392 5610	282 (14.1%) 185 (3.3%)	312 (15.6%) 410 (7.3%)	604 (30.2%) 1387 (24.7%)	501 (25.1%) 1966 (35.0%)	298 (14.9%) 1662 (29.6%)	<0.001
Food insecurity Yes No	1449 6158	115 (7.9%) 352 (5.7%)	177 (12.2%) 545 (5.7%)	484 (33.4%) 1507 (24.5%)	422 (29.1%) 2045 (33.2%)	251 (17.3%) 1709 (27.8%)	<0.001
Hypertension Yes No	2528 5035	172 (6.8%) 295 (5.9%)	270 (10.7%) 448 (8.9%)	675 (26.7%) 1304 (25.9%)	768 (30.4%) 1685 (33.5%)	643 (25.4%) 1303 (25.9%)	0.009
Asthma Yes No	585 380	45 (7.7%) 21 (5.5%)	62 (10.6%) 39 (10.3%)	160 (27.4%) 104 (27.4%)	180 (30.8%) 130 (34.2%)	138 (23.6%) 86 (22.6%)	0.64
Hypercholesterolemia Yes No	2270 3051	114 (5.0%) 186 (6.1%)	221 (9.7%) 278 (9.1%)	569 (25.1%) 734 (24.1%)	729 (32.1%) 991 (32.5%)	637 (28.1%) 862 (28.3%)	0.43
Diabetes Yes No	892 6715	64 (7.2%) 403 (6.0%)	120 (13.5%) 602 (9.0%)	277 (31.1%) 1714 (25.5%)	253 (28.4%) 2214 (33.0%)	178 (20.0%) 1782 (26.5%)	<0.001
Rheumatoid Arthritis Yes No	401 1617	34 (8.5%) 110 (6.8%)	47 (11.7%) 178 (11.0%)	109 (27.2%) 357 (22.1%)	101 (25.2%) 464 (28.7%)	110 (27.4%) 508 (31.4%)	0.09
BMI, kg/m^2^	7607	29.0 ± 7.25	29.9 ± 7.42	29.8 ± 6.80	28.6 ± 6.08	27.1 ± 5.50	<0.001
CRP, mg/dL	7607	0.67 ± 1.44	0.61 ± 1.22	0.53 ± 0.88	0.45 ± 0.91	0.37 ± 0.60	<0.001
RDW	7607	13.0 ± 1.49	12.9 ± 1.34	12.9 ± 1.25	12.8 ± 1.11	12.7 ± 1.09	<0.001
CAD Yes	701	67 (9.6%)	84 (12.0%)	173 (24.7%)	202 (28.8%)	175 (25.0%)	<0.001
No	6906	400 (5.8%)	638 (9.2%)	1818 (26.3%)	2265 (32.8%)	1785 (25.8%)	

Abbreviations: PIR, Poverty to Income Ratio; BMI, body mass index; CAD, coronary artery disease; CRP, C-reactive protein; PIR, poverty to income ratio; RDW, red cell distribution width.

**Table 3:** Logistic Regression Results Predicting the Probability of CAD from Vitamin C Categories.

Predictor	Unadjusted OR (CI) ^a,b^	Adjusted OR (CI) ^d,e^
Gender Male Female	1.31(1.05, 1.62) ^c^ Ref	1.33 (0.62, 2.82) Ref
Adulthood Stage Young, 20–39 Middle, 40–59 Late, ≥60	Ref 7.49 (4.24, 13.2) ^c^ 43.2 (25.0, 74.7) ^c^	Ref 1.64 (0.18, 14.9) 3.35 (0.38, 29.6)
Race/Ethnicity Mexican American Other Hispanic Non-Hispanic White Non-Hispanic Black Other	0.58 (0.43, 0.80) ^c^ 0.42 (0.17, 1.01) Ref 0.93 (0.71, 1.20) 1.29 (0.72, 2.27)	0.50 (0.11, 2.29) 1.42 (2.64, 7.80) ^c^ Ref 0.16 (0.04, 0.56) ^c^ 6.29 (1.01, 39.2) ^c^
Family PIR High Medium Low	0.94 (0.70, 1.27) 1.27 (0.85, 1.62) Ref	1.58 (0.43, 5.90) 2.83 (0.70, 11.5) Ref
Smoking status Yes No	1.11 (0.85, 1.46) Ref	1.12 (0.86, 1.47) Ref
Food insecurity Yes No	2.11 (1.58, 2.80) ^c^ Ref	2.06 (1.54, 2.75) ^c^ Ref
Vitamin C Deficiency Hypovitaminosis Inadequate Adequate Saturating	2.31 (1.49, 3.58) ^c^ 1.46 (1.00, 2.12) 1.39 (1.03, 1.87) ^c^ 1.16 (0.88, 1.54) Ref	1.62 (0.47, 5.51) 1.23 (0.42, 3.64) 0.33 (0.13, 0.86) ^c^ 1.21 (0.52, 2.82) Ref
Hypertension Yes No		2.33 (0.89, 6.10) Ref
Hypercholesterolemia Yes No		1.48 (0.63, 3.26) Ref
Diabetes Yes No		3.14 (1.40, 7.07) ^c^ Ref
Asthma Yes No		1.24 (0.48, 3.20) Ref
Rheumatoid Arthritis Yes No		1.14 (0.34, 3.83) Ref
BMI, kg/m^2^	1.03 (1.01, 1.04) ^c^	0.90 (0.85, 0.95) ^c^
CRP		1.22 (0.84, 1.76)
RDW		1.68 (1.14, 2.46) ^c^
Constant	166.9 ^c^	5.89 ^c^

Abbreviations: BMI, body mass index; CAD, coronary artery disease; CRP, C-reactive protein; OR, odds ratio; PIR, poverty to income ratio; RDW, red cell distribution width; Ref, reference. ^a^ Model (likelihood ratio) χ^2=^ 25.7, df = 15, *p* < 0.001. ^b^ Nagelkerke R^2^ = 0.23. ^c^ *p* = <0.05. ^d^ Model (likelihood ratio) χ^2^= 31.7, df = 20, *p* < 0.001. ^e^ Nagelkerke R^2^ = 0.35.

## Data Availability

The publicly available data sets used in this study can be found on the Centers for Disease Control and Prevention (CDC) website at: https://www.cdc.gov/nchs/nhanes/index.htm (accessed on 5 March 2022).
